# Sustainable Utilization of Traditional Chinese Medicine Resources: Systematic Evaluation on Different Production Modes

**DOI:** 10.1155/2015/218901

**Published:** 2015-05-17

**Authors:** Xiwen Li, Yuning Chen, Yunfeng Lai, Qing Yang, Hao Hu, Yitao Wang

**Affiliations:** ^1^Research Center for Pharmacognosy, Institute of Chinese Materia Medica, China Academy of Chinese Medical Sciences, Beijing 100700, China; ^2^Institute of Medicinal Plant Development, Chinese Academy of Medical Sciences, Peking Union Medical College, Beijing 100193, China; ^3^State Key Laboratory of Quality Research in Chinese Medicine, Institute of Chinese Medical Sciences, University of Macau, Macau; ^4^State Key Laboratory of Hydraulics and Mountain River Engineering, Sichuan University, Chengdu 610065, China

## Abstract

The usage amount of medicinal plant rapidly increased along with the development of traditional Chinese medicine industry. The higher market demand and the shortage of wild herbal resources enforce us to carry out large-scale introduction and cultivation. Herbal cultivation can ease current contradiction between medicinal resources supply and demand while they bring new problems such as pesticide residues and plant disease and pests. Researchers have recently placed high hopes on the application of natural fostering, a new method incorporated herbal production and diversity protecting practically, which can solve the problems brought by artificial cultivation. However no modes can solve all problems existing in current herbal production. This study evaluated different production modes including cultivation, natural fostering, and wild collection to guide the traditional Chinese medicine production for sustainable utilization of herbal resources.

## 1. Introduction

Traditional Chinese medicine (TCM) recently is widely accepted by patients and attracting more and more attention of researchers with the change of disease modes and the rise of “return to nature” in the world. Currently 45% of all countries (regions) in the world are using TCM, and the global trade of TCM has reached 40 billion USD a year, with an increasing rate of 10% per year [1]. China exported 25% of the global demand of TCM while 75% of which was raw materials [2]. Currently, a total of 80% of TCM are from continuous wild collection without scientific plans [3]. The natural reserves can hardly meet the rapidly increasing demand. At the same time, the wild herbal resources are quickly decreasing by 30% every year. Consequently 80% of the most usually used species cannot meet medical demand [4]. Data analysis showed that 1,800–2,100 medicinal species were facing the challenge of extinction in China [5]. Even though some of the wild herbs can recover naturally within 3–5 years, the recovery speed falls much behind the one of the rising demand. As a result, TCM resources are facing more and more challenges of sustainable utilization.

Long-term sustainable utilization of TCM resources should combine market demand of raw materials, ecological stability, and social benefits [6]. Currently cultivation and natural fostering were the main production modes to ease contradictions between the decrease of natural reserves and the increase of market demand of wild medicinal resources [7]. Cultivation can be implemented in a large scale and is an efficient method to rapidly provide sufficient urgent raw medicinal materials compared with other methods. However cultivation requires massive cultivated land and plant disease and pests, heavy metal and pesticide residue are the major obstacles to limit its application to all medicinal plants. Moreover the quality of its output (raw materials) sometimes cannot satisfy the clinical criteria. Natural fostering, also named as wild nursery or semi-imitational cultivation, is a new kind of herb production method. It can combine economic benefit and diversity protecting practically and can solve the problem between subsistence and biodiversity conservation effectively. In China emphasis is given to natural fostering which aims to maintain and recover viable populations of wild species in nature. But it has still limitations such as the long-time process and low output, which cannot meet the rapid increase of market demand in short term. Though wild collection, natural fostering, and cultivation own advantages in yielding raw medicinal materials, they cannot completely solve the current problem of herbal resource sustainable utilization alone. How to choose production mode depends on natural reserves, usage amount, and biological characteristics of medicinal plant species. This study performs a systematic evaluation on these three production modes in different dimensions including current application status, technological challenges, input-output ratio, and ecological impact. In addition, we present illustrations to indicate the characteristics of each method, which can practically guide the selection of TCM production modes for resource sustainable utilization.

## 2. Current Status of Different Production Modes

### 2.1. Wild Collection

TCM resources mean the healing herbal materials. According to the statistics, there are 11,146 medicinal plants species, belonging to 383 families, and 2,313 genera [8]. The herbal geographical distribution covers different longitude, latitude, and altitude in China. Different ecological habitat causes different genuine medicinal materials. Nowadays there were 100,000 traditional Chinese medicine prescriptions and these prescriptions used 700 Chinese herbal species, 80% of which comes from wild collection [9] such as* Polygonum cuspidatum*,* Leonurus japonicus*,* Forsythia suspensa,* and* Bupleurum chinense*.  Table 1 summarizes the mostly used Chinese herbs that need wild collection. Due to the finite herbal storage, the increasing demand, and the harsh living conditions, the output of wild herb collection is reducing every year.

Even though the Chinese government has started to improve ecological environment to protect the Chinese herbal habitat, the increasing demand of the whole world market still makes a great deal of medicinal plants face the possible extinction. The problems including the lack of wild herbal collecting plan, biomes' destruction, and degraded ecologic environment are becoming more and more serious. Some famous wild herbs, such as* Cordyceps sinensis*,* Dendrobium officinale*,* Fritillaria cirrhosa,* and* Saussurea involucrata*, are becoming more and more difficult to be found in wild habitat. Although some medicinal plants have been successfully cultivated, their wild species can still no longer be found within the latest decades of years, just like* ginseng*,* notoginseng*, and* Gastrodiae*. It would lead to great obstacles in future when these genuine medicinal plants need to be selected from wild resources for breeding.

The main reasons that endanger the wild Chinese herbal resources are the following. Firstly, the national and international market demand is boosting. There are 1.35 billion people in China in 2014. This is a huge consumption group. Besides, Chinese herbal trade has extended to 120 countries in the world market. The herbal varieties have reached 500 species and mainly were transported abroad as raw materials [10]. Secondly, the worsening global climate and Chinese ecological environment are threatening medicinal plant habitat. In the past 30 years the rapid industrialization of China caused a huge pressure to wild environment. The changing environment lowered the recovery speed of wild herbal plants. Some herbal plants even cannot be recovered at all because of the damage of their natural environment. Thirdly, wild collection was not scientifically carried out. In China the people who work on wild herb collection are almost the peoples with a low education level. Most of them have little knowledge on herbal sustainable utilization. They tended to follow their own habit and economic motivation to collect wild herbs, which caused that some herbs and their habitats were destroyed destructively. For example, digging a wild licorice has to destroy accompanying plants of 10 m^2^. And digging up wild plants of* Ephedra* species destroyed 3,200 hm^2^ meadow every year [11]. Therefore current wild collection cannot guarantee the sustainable development of Chinese herbal resources.

### 2.2. Natural Fostering

Natural fostering mainly focuses on increasing the number of herbal population to provide raw medicinal materials. This method should be implemented in original habitats, which is different from artificial cultivation. Natural fostering can effectively combine medicinal plant production and economic benefit. It can increase the recovery ratio of original population. Finally, it does not change the basic community trait of original habitat. Therefore, natural fostering unites industrialized production of TCM and ecological protection. Natural fostering has been carried out in many Chinese herbs, such as* Fritillaria cirrhosa*,* F. unibracteata*,* Glycyrrhiza uralensis*,* G. inflate*,* G. glabra*,* Panax ginseng*,* Ephedra sinica*,* E. intermedia*,* Coptis chinensis*,* C. deltoidea*,* C. teeta*,* Gastrodia elata*,* Saussurea involucrata*, and* Cordyceps sinensis*. Practice proved it to be a pragmatic way to produce TCM materials and conserve biological diversity.

Natural fostering is mostly suitable for such herbs. Firstly, their original habitats are special and cultivation cost is very high. Secondly, their commercial characters and quality have great variations after being cultivated. Thirdly, their wild distribution areas are concentrated and great achievement of production will be made by natural fostering.

Natural fostering is an innovative method for Chinese herbal production. More than 19 Chinese herbs have been used to produce raw materials through natural fostering (Table 2) and among which 12 herbs have realized large-scale production. The key advantage of natural fostering is that the herbal quality from its output is very close to that from wild collection. For example, the polysaccharide content of wild* Ranunculus ternatus* was 14.1% and 10% from natural fostering. However the content of total amino acids of* Ranunculus ternatus* was 2-3% higher from natural fostering than from wild collection [12]. But they almost had the same kinds of amino acids. Similarly, wild* Cordyceps sinensis* had the same varieties of amino acids as that from natural fostering and both of their adenosine contents are more than 0.1 mg/g, which meet the quality requirement of Chinese Pharmacopoeia [13]. The profit motivation was the main drive to prompt peasants to implement natural fostering of medicinal plants. For example, the income from fostering* Coptis* was 15 times higher than the one from planting crops [14].

Natural fostering is a promising herbal producing method which is a combination of wild collection and cultivation [6]. But its technology is still not mature and the germ plasm for natural fostering has not been identified completely. The production scale is not as large as artificial cultivation and stays at the primary phrase. Although there were numbers of experimental project of natural fostering in China, they were limited by the weakness of basic studies and long-term process. The success of natural fostering also depends on the further study of specie characteristics.

### 2.3. Cultivation

Herbal cultivation is one of the most effective methods which can not only satisfy market demand but also release the ecological pressure caused by wild collection. In China, the area of herbal cultivation has increased from 400,000 hm^2^ in 1950s to current 9,330,000 hm^2^. There were altogether 200 herbs that can be artificially cultivated, 100 of which have achieved large-scale cultivation including* Eucommia ulmoides*,* Magnolia officinalis*,* Bupleurum chinense,* and* Platycodon grandiflorum* [15]. The output of herbal production by cultivation has reached 400,000 tons per year. The yield of 200 usually used Chinese herbal medicine from cultivation accounted for more than 60% of the whole market demand per year in China. In particular some herbs such as* ginseng* and* notoginseng* were provided absolutely by cultivation. More and more companies are beginning to recognize the supply crisis of raw medicinal materials, and the Good Agriculture Practice (GAP) was implemented widely.  Table 3 summarized the main artificially cultivated herbal species.

TCM cultivation not only provides raw medicinal materials but also brings additional essential problems such as excessive heavy metal and pesticide residues. Although the number of herbs which can be cultivated is increasing, artificial breeding has been carried out on only 20 kinds of herbs [16]. The degeneration of germ plasm leads to plant diseases and insect pests and significant output reduction. It was estimated that the planting area in 2008 decreased by 40% compared with the area in 2002. Plant diseases and insect pests were one of the main reasons [17, 18]. Another problem of herbal planting was the lack of scientific design due to profit issues. When planting fruits, crops, and economic forest can bring more income than planting herbs, farmers will give up herbal cultivation. For example, the scale of* Polygonum multiflorum* in 1977 had reached 453.3 hm^2^ in Deqing County in Guangdong Province while the planting area decreased by 90% in 2012 because planting citrus and other fruits can bring more income.

Although herbal cultivation can increase production and economic profit rapidly in single population, it is not a sustainable way for the development of herbal resources. Compared with crop cultivation which aims to get the first metabolite (protein, fat, sugar, etc.) of the plants, the main purpose of herbal production is to produce the secondary metabolites (such as alkaloids, saponins, terpenes). Herbal cultivation goes against plant natural growing regularity by escaping from community environment. It is suggested that improving cropping ratio and intercropping ratio may be able to solve the current problems in herbal cultivation.

## 3. Technological Challenges

### 3.1. Wild Collection

The key technological points of wild collection include collection methods, transportation, species identification, and collecting period. Among these factors, one of the most crucial technological difficulties is collection method. We should carefully design collection method to avoid possible damage on surrounding plants. At the same time, another difficulty of wild collection is transportation. The original distribution of wild medicinal plants is usually located in remote mountainous areas with poor establishments whose inconvenient traffic and poor information systems make it challenged to transport the wild collected herbs [7]. For example, wild liquorice, ephedra, and* Cistanche* mainly distribute in desert areas and are difficult to be transported. Moreover, long transportation process may also have influence on the quality of Chinese materia medica.

### 3.2. Cultivation

Germ plasm selection, breeding, fertilization, and prevention of diseases and insect pests should be paid more attention to in herbal cultivation.

#### 3.2.1. Fertilization

Currently few basic studies on agrology of TCM were systematically carried out. We still do not know much about suitable soil conditions, balanced fertilization technology, and the relationship between soil environment and herbal intrinsic quality [19]. Fortunately, the varied soil conditions are being gradually recognized. Researchers found that, in the 29 cultivation areas of* Paris polyphylla* Smith var.* yunnanensis* in nine cities of Yunnan Province, the nutritional soil status was extremely uneven. Soil nitrogen in eight of these cultivated areas was below the normal level, which would affect herbal growth and output in the next year. The absorption of phosphorus and potassium was also influenced. The pH value of four cultivation areas was beyond the optimal growth range (4.5 to 6.3) [20]. People gradually realized that the rhizome of some herbs has the ability to enrich heavy metal elements. Even though the content of heavy metals was in a low level in soil, the actual amount of heavy metal in herbal materials still exceeded the standard. Han et al. found that the average content of copper, lead, arsenic, cadmium, and mercury exceeded the standard 21.0%, 12.0%, 9.7%, 28.5%, and 6.9%, respectively, after analyzing 312 kinds of Chinese herbs [21].

#### 3.2.2. Pest Prevention

The basic researches on pest prevention of Chinese herbal species remained limited until recently. The studies on the relationship among soil herbs and germ, as well as the physiological, biochemical, and molecular mechanic researches, were also in infancy. Because there are many differences between Chinese herbal species and crops including growth habit, stress resistance, and main target products, Chinese herbal cultivation cannot just apply the techniques used in ordinary crops completely. According to an investigation analyzing 300 kinds of Chinese herbal materia medica, the majority of the samples had residue of organic chlorine pesticides which can lead to hepatomegaly, degeneration of liver cell, damage of central nervous system, and bone marrow [22].

#### 3.2.3. Seed Selecting and Breeding

Improper selection of original herbal species will lead to species confusion and reduction of diversity. For example, seeds from all kinds of* Cannabis* can be used as Huomaren materia medica, which causes the confusion of Huomaren germ plasm. Due to the divergence of stress resistance and growth habit, it is great difficult to introduce and cultivate* Cannabis sativa* according to the same protocol [23]. The most difficulty to select medicinal varieties was the intraspecies variation. Different classifications were divided according to the variation including subspecies, variety, and variant, which caused the quality divergence of herbal medicine and different clinical efficacy [24]. It is difficult to find good variety possessing not only the highest yield but also the best quality. Unfortunately there have been no new varieties with stable genetic property up to now.

### 3.3. Technological Challenges for Natural Fostering

The key technological points of natural fostering include selection of site, construction of seedlings base, density adjustment, and scientific harvesting. Among these technological points, the main challenges for natural fostering include the following.

#### 3.3.1. Selection of Site

Natural fostering requires that the site should be in the original habitat or the ecological conditions similar to the original habitat areas. To determine whether an area is suitable for breeding, the most reliable and effective way is to carry out a longer period of experiment. However, it needs a lot of manpower, material, and observation for up to several years of growth cycle, and in practice the experiment is much difficult to carry out in a large scale [25]. A wild habitat may not be suitable for the growth of target medicinal species; at least most of the plants cannot be guaranteed to live in optimal living conditions and toward the development of population growth. So it is difficult to develop natural fostering to reach a large industrial scale in some situations [26]. Multicriteria assessment is essential to select suitable fostering site: (1) direct information on species distributions; (2) market analysis on potential medical plants; (3) community types or biotic units according to the evaluation of effective components; (4) transportation convenience; and (5) other goals.

#### 3.3.2. Construction of Seedlings Base

The key part of base construction is how to produce vigorous seedlings or healthy seeds. Original funds and technologies are very important. The aims of nursing seedlings are somewhat different from breeding in agriculture. Two methods are usually adopted: selection of germ plasm resources and crossbreeding. Selections of germ plasm resources are from wild species whose characters include ability of resisting adversity, high production, high content of effective chemical component, and so on. Sometimes, the selection needs to be adjusted in terms of relative medical aims such as medicinal organs (flower, radix, rhizome, and leaf), characters of effective component (volatile, poisonous), and values of medical goods (“daodi”, shape). Crossbreed can be operated according to common approaches in agriculture [26]. One point needs to be announced that medical raw materials from crossbreed have not been verified through long-term experiments and not yet accepted by traditional Chinese herbalist doctors. From this view, crossbreed in natural fostering is used in yielding of medical materials for component of extraction, not for “yinpian” (semimanufactured goods for medicine through different physical methods). In addition, building and field construction is absolutely necessary [27].

#### 3.3.3. Scientific Harvesting

Three parameters should be taken into account in natural fostering: quantity to harvest, time of harvest, and the condition of the plant community. The quantity of picking should not affect community structure and not pick in excess to ensure sustainable utilization in following years. It is absolutely necessary to pick medicinal materials in proper time because the content of effective chemistry component is different in different stages. As the extension of fostering time, herbal population density, medicinal ingredients, and biomass gradually increase. However, natural fostering may cause the degradation of competitiveness in licorice population after 5 years [28]. For some herbs, the shorter the growing years, the higher the content of the active ingredient such as calycosin glucoside and formononetin in* Astragalus membranaceus* [29]. Although early acquisition of these herbs could get better economic benefits, it may bring ecological loss. On the contrary, for some herbs the longer the growing years, the higher the content of medicinal ingredient such as ginseng. In order to achieve bigger production and high content of component, studies should be carried out on content dynamic curves of aimed medicinal materials in its life. The final objective is to find out key point of intersection between quantity and content to get the biggest effective biomass. All of the operations should preserve ecological stability, after all, which is our objective.

## 4. Economic Input and Output

### 4.1. Wild Collection

Wild collection depends on directly picking raw medicinal materials from natural resources. Therefore manpower is the main economic input. Local people do not need to invest any other economic resources to conduct wild collection, which generates great motivation for wild collection without considering systematic plan and the damage of ecological environment. Li et al. found that the annual income of farmers in Zhouzhi Country was 1,767.7 RMB/household in 2007 only by wild herbal collection [30]. Another study found that the annual income was 1,200 RMB/person in Ussuri area of Heilongjiang Province, which stimulated more than 3,000 persons to join in wild herbal collection [31].

### 4.2. Cultivation

The economic input of herbal cultivation includes land leasing, buying seeds, irrigation, fertilizers, pesticides, and manpower. Generally, the bigger the planting area, the higher the yield, the higher the economic income, and the higher the land cost. Most of leasing lands are located in the main producing areas, and the rents are different among different regions. According to the statistics of the National Agricultural Cost-benefit Data Assembly, the land rents of ginseng in Jilin, Liaoning, and Heilongjiang Province were 9.07 RMB/m^2^, 0.54 RMB/m^2^ and 0.46 RMB/m^2^, respectively. Moreover, the land rents of cultivating different herbs in the same province were also different. For example, in Hubei Province the average land rent of* Coptis* was 0.14 RMB/m^2^ but 0.07 RMB/m^2^ for Kikyo. In addition, different planting methods also result in different output-to-input ratios. As shown in Tables 4 and 5, the input-outcome differences between the standard planting (demonstration group) and nonstandard planting (control group) for* Pinellia* are obviously apparent [32].

Comparing the two planting methods, there were significant differences between the cost of seeds, organic fertilizer, and employee. And the output-to-input ratio of standard planting methods was higher than the one of nonstandard planting. It indicated that more attention should be paid to seeds, soil management, and fertilization in herbal cultivation.

### 4.3. Natural Fostering

The economic input of natural fostering includes buying seeds, base construction, and harvest. Different fostering modes can bring different economic benefit.  Table 6 showed that planting* Coptis* using three models (cultivation in greenhouse, fostering under* Cryptomeria japonica* and* Coriaria nepalensis* forest, resp.) caused different output-input ratios. Cultivation in greenhouse required the highest cost and fostering under* Cryptomeria japonica* forest brought the highest yielding. Natural fostering has a great advantage in planting Coptis. As shown in Table 7, it is proved that natural fostering had higher benefit than cultivation in ginseng production (data from [33]). Compared with herbal cultivation, natural fostering was mainly implemented in mountainous areas, which caused higher cost of employees due to the poor traffic. As the examples of* Coptis* and* Rhizoma Paridis* fostering in Dujiangyan County of Sichuan Province, the human cost of transportation is 170 RMB from picking point to collection area [34]. Therefore, Chinese medicine enterprises have to build their factories close to the fostering area to reduce transportation cost.

## 5. Ecological Impact

### 5.1. Wild Collection

Wild collection generated great influence on ecological environment and biodiversity. Overexploitation had a damaging effect on plant vegetation, which made the surface exposed to air and led to land desertification and the subsequent severe soil loss. In addition, excessive wild collection destroyed community structure and the ecological balance. One of the main reasons for the ecological system imbalance of Inner Mongolia grassland was due to the excessive digging of wild licorice. The natural recovery of plant vegetation in the northwest arid areas was becoming more and more difficult. It has formed a vicious cycle of ecological problems among vegetation deterioration, soil erosion, resource exhaustion, and regional poverty.

Due to the deterioration of ecological environment by human impact, the amount of wildlife resources has been reducing while the amount of endangered species continued to increase. It is estimated that 15,000 of 72,000 species of medicinal plants in the world have been endangered by reason of overexcavation [35]. In China, there were 1,800–2,100 of 11,146 species of medicinal plants that have been endangered and 20% of all commonly used herbs were facing shortage [9]. The main endangered reason of medicinal plants was due to the lack of regulation and scientific planning in wild herbal collection. Typically,* Taxus chinensis* now was at the verge of extinction as the massive deforestation destruction when anticancer function of its main medicinal ingredient (taxol) was found in 1967.

Since the rapid development of the Chinese herbal medicine industry, wild herbal resources have suffered a predatory excavation. Furthermore, some herbal resources will still remain scarce, endangered, or extinct within a considerable period of time. For example, the natural reserve of wild* Lamiophlomis rotata* was 3,713–6,896 tons, the maximum allowable annual yield was about 1,700 tons, and the recovery period was 4-5 years. However, the actual amount of annual collection is about 2,520 tons. It would be unable to achieve sustainable utilization of wild* Lamiophlomis rotata* if picking keeps the current amount [36].

### 5.2. Cultivation

The shortage of wild medicinal resources and the development of modern Chinese medicine enterprises forced people to introduce and cultivate herbal medicine. But herbal cultivation has brought new ecological and environmental issues caused by deforestation, pesticide residues, and heavy metal pollution. Meanwhile original plant vegetation and secondary forests were inevitably destroyed more or less when cultivation of Chinese herbs was widely adopted. The ecosystem balance is facing the risk of severe damage by large-scale planting medicinal plants. In addition, soil structure was broken such as compaction due to excessive use of fertilizer. The phenomenon of soil heavy metal pollution is getting worse with the overuse or misuse of pesticides because of the lack of basic knowledge of diseases and pest control. It is common to see that both plant insect pests and their predators are killed because some current commonly used pesticides are too toxic. Sometimes several violent poisons were mixed to prevent or kill insect pests in order to achieve a quick and effective purpose. Although the yield of materia medica has been improved, they could not be used for clinical medicine. The final result was that environment became worse and the quality of Chinese herbal medicines could not meet standards [37]. Yi and Lu [38] found that pesticides and heavy metals not only existed in herbal medicines but also remained in soil.

### 5.3. Natural Fostering

The key point of natural fostering is not only to increase population density but also to involve human intervention as little as possible. Compared with cultivation, it can greatly reduce economic input and can provide Chinese herbal raw materials with high quality without influencing natural environment. In the process of fostering* Fritillaria* in Zheduo Mountain, there were 6 community types of wild* Fritillaria* and more than 40 kinds of main accompanying species which greatly exceeded the control areas. In addition, the biodiversity in fostering regions is also being increased [39]. Natural fostering in wild licorice made great achievements by using fence to limit excessive excavation in Hangjinqi area of Inner Mongolia. The coverage of licorice reached 63.3% and the continuous cover area reached 4,000 hm^2^, which can be served as a barrier to prevent windstorms and fasten sand in the land [40].

Forest breeding is another kind of natural fostering. In the past 300 years,* Coptis* was planted under simple shacks that were built in the mountains without trees. In this way, all the trees and weeds were removed, and even the roots in soil must be cleared. Both the topsoil and network structure of the soil were destroyed, which can easily cause soil loss. Generally, a total of 3 m^2^ forests need to be cut down for cultivating 1 m^2^ *Coptis* [41]. On the contrary,* Coptis* production using natural fostering under forest not only improved the yield but also increase the forest coverage [42]. Natural fostering can increase the ability of community resilience. It integrates herbal medicine production with community conservation to address three crucial problems simultaneously, namely, funding, public participation, and rural living [40]. The logistic relationships among them can be explained in the following perspectives: (a) the raw materials of traditional medicine are largely derived from herbal medicine; (b) herbal medicines of high quality are closely correlated with the physical and chemical environments of communities; (c) only materials derived from intact communities are of high quality and may be sold at high prices; (d) Local communities will take the initiative to preserve community integrity so that they are able to harvest high quality natural products for improving income; (e) local people with surplus money can invest on community integrity conservation, which results in a good cycle.

## 6. Conclusion and Perspectives

As a result of the increasing demand for medicinal plants, most of which is still met by wild collection, a constant pressure is created on existing resources, leading to continuous depletion of some of the species in the forests, and at the same time forest land is losing its natural flora at an alarming rate. Although the shortage of medicinal materials is alleviated to some extent since more than 200 kinds of Chinese herbs could be artificially planted, the new problems of variety degeneration, pesticide residues, and the heavy metal pollution caused by cultivation under single species population have forced to seek more innovative methods. Researchers have placed high hopes on natural fostering to make up the deficiency of wild collection and artificial cultivation. In particular, it has little impact on ecological environment and can produce raw herbal materials with a considerable high quality, also as an approach to conserve biological conservation. But it cannot completely replace the other two methods because of the long production cycle and the technological imperfections. More attention has been directed on the value of combined method from a holistic perspective, exploring the feasibility to solve the conflict between biodiversity and economy. Therefore, the sustainability of Chinese herbal resources should depend on systematic combination of wild collection, cultivation, and natural fostering, with comprehensive consideration of medical demand and herbal growth characteristics.

## Figures and Tables

**Table 1:** The yield of mostly used Chinese materia medica through wild collection.

Materia medica	Output (10^7^ kg)	Main producing areas	Reference
Huzhang	1	Huaihua, Jingmen	[43]
Jixueteng	0.5–1	Lincang, Simao, Dehong, Yulin	[43]
Yimucao	0.5–1	Tanghe, Zaoyang, Huxian	[43]
Lianqiao	0.6	Lushi, Yuncheng, Xinjiang	[44]
Caihu	0.6	Li and Dangchang in Gansu Province; Chengcheng and Wenxi in Shanxi Province	[45]
Chonglou	0.3	Qujing, Dali, Lijiang, Yuxi, Zhaotong, Chuxiong	[46]
Changzhou	0.28–0.3	Acheng, Wuchang, Bingxian, Huadian, Tonghua, Yongji	[47]
Duyiwei	0.25	Gannan, Yushu; Yunnan, Sichuan; and Tibet Province	[36]
Guanfangfeng	0.15	Heilongjiang, Jilin, Liaoning Province, Inner Mongolia, Hebei, and Shandong Province	[48]
Baiji	0.85	Anlong, Xingyi, Duyun, Neijiang, Wenjiang	[49]

**Table 2:** Main Chinese materia medica under natural fostering in China.

Chinese herb	Location	Area (km^2^)	Reference
Chuanbeimu	Kangding	20	[50]
Gancao	Yanchi, Lingwu, Tongxin, Taole	49	[51]
Huanglian	Fugong district in Nujiang	1	[14]
Mahuang	Inner Mongolia Alukerqin	1000	[52]
Wuweizi	Qingyuan, Xinbin, Fengcheng	32	[53]
Luobuma	Bazhou in Xinjiang Province	—	[54]
Ciwujia	Cuiluan district in Yichun city	—	[55]
Huangqi	Along the Golmud section of the Qinghai-Tibet Railway to Amdo	—	[56]
Shizhushen	Kuandian district in Dandong	0.2	[57]
Zhuling	Neiqiu County	—	[58]
Yinyanghuo	Leishan, Xiuwen and Longli County	27	[59]
Jinlianhua	Weichang County in Hebei Province	20	[60]
Bajiaolian	Nanchuan, Chongqing	—	[61]
Tangbanxia	Tanghe County	1.25	[62]
Lianqiao	Zhongtiao Mountain	6.7	[44]
Xuelian	Yili city	0.02	[63]
Roucongrong	Hetian in Xinjiang Province	129	[64]
Zhongjiefeng	Guangzhou	—	[65]

**Table 3:** Main Chinese materia medica under cultivation.

Chinese materia medica	Main producing area	Area (km^2^)
Dangshen	Linchuan	3.3
Danshen	Shangluo, Fangcheng, Zhongjiang, Linqi	51
*Panax pseudoginseng *	Wenshan	67
Banlangen	Fuyang, Daqing	40
American ginseng	Jingyu	10
Panax ginseng	Jingyu, Fusong, Ji'an	10
Coptis	Shizhu, Zhenping, Enshi city	45
Huajuhong	Huazhou	10
Xuanshen	Zhenping	7
Changzhou	Luotian	2
Touhualiao	Shibing	20
Ginkgo	Chongming, Pizhou	24
Jinyinhua	Pingyi, Fengqiu	12
Tiepishihu	Wuyi, Tiantai, Xinshuangbannan	6
Chuanxiong	Pengzhou	67
Dihuang	Wushe	200
Fuzi	Jiangyou	2
Shanmaidong	Quanzhou	5
Chuanxinlian	Qingyuan	3
Dengzhanhua	Luxi	7
Chuanbei	Songpan and Mao County	2
Shanzhuyu	Xixia County	147
Yanhusuo	Fuzhou in Jiangxi province	24
Kushen	Changzhi	67
Longdan	Qingyuan	13

**Table 4:** The cost of *Pinellia* production (RMB).

	Seed		Calcium fertilizer		Urea		Organic fertilizer		Pesticides	Employees		Equipment	Total
	Amount	Price	Amount	Price	Amount	Price	Amount	Price	Total	Amount	Price	Price	
Standard	2,250	36,000	1,500	1,050	225	450	45,000	4,500	150	480	14,400	2,100	58,650
Control	2,250	27,000	1,500	1,050	225	450	37,500	3,750	150	480	12,600	2,100	47,100
Difference		9,000		0		0	700	750	0	60	1,800	0	11,550

**Table 5:** The output-input ratio in *Pinellia* production.

	Output (kg per hm^2^)	Value (RMB per hm^2^)	Benefit (RMB per hm^2^)	
			Net income	Input : output
Standard	3,093.43	134,564.21	75,914.21	1 : 2.294
Control	2,279.25	99,147.38	52,047.38	1 : 2.105
Difference	814.18	35,416.83	23,886.83	1 : 3.066

**Table 6:** The cost under different *Coptis* production modes (RMB).

Model	Outcome	Input	Net income	Input : output
Cultivation in greenhouse	14,730	7,100	7,630	1 : 2.075
Natural fostering under *Japanese cedar* forest	14,250	4,800	9,450	1 : 2.968
Natural fostering under *Coriaria nepalensis* forest	13,605	4,800	9,005	1 : 2.834

**Table 7:** Comparison between different models of ginseng production (10,000 RMB/hectare).

	Input	Output	Cost-profit ratio	Net margin
Natural fostering	11.5	23.3	1.026	11.8
Cultivation	16.4	22.0	0.335	5.6

## References

[1] Yang W. (2008). Development situation of China's traditional Chinese medicine export trade and its countermeasure study. *Guangdong Trace Elements Science*.

[2] Jia J., Jingping L. (2011). The position, problem and tactic of Chinese medicine industry. *Guangdong Agricultural Sciences*.

[3] Shilin C., Peigen X. (2006). *Introduction to the Sustainable Utilization of Chinese Herbal Medicine Resource*.

[4] Zhuyun Y. (2012). Major tasks and challenges for resources science of Chinese Medicinal Materials. *Pharmacy and Clinics of Chinese Materia Medica*.

[5] Huang L., Guo L., Cui G. (2005). Basic theory research of traditional Chinese medicine resources. *Research and Information on Traditional Chinese Medicine*.

[6] Chen S.-L., Su G.-Q., Zou J.-Q., Huang L.-F., Guo B.-L., Xiao P.-G. (2005). The sustainable development framework of national Chinese medicine resources. *China Journal of Chinese Materia Medica*.

[7] Gan T. (2009). Economic analysis of wild medicinal material depletion. *Rural Economic*.

[8] Hai Y., Taikang H., Chunfu W. (2005). Processes and trends in the development of modern medicine. *Chinese Traditional and Herbal Drugs*.

[9] Huang L., Peng H., Xiao P. (2011). Development trend of traditional Chinese medicine resources. *China Journal of Chinese Materia Medica*.

[10] Zhisheng M., Wensheng Z., Yongyan W. (2008). The protection of wild herbs need to be improved in China. *Chinese Journal of Information on TCM*.

[11] Zhang Y., Si J., He B. (2002). Present situation of ephedra resources and their exploiting countermeasures. *World Science and Technology/Modernization of Traditional Chinese Medicine*.

[12] Chi C., Kaijia H., Buming L. (2008). The study on the comparison between wild and cultivated radix ranunculus ternatus. *Guangxi Sciences*.

[13] Chen S., Yun D. (2003). Comparison of semi-wild and wild Cordyceps chemical composition. *Journal of Chinese Medicinal Materials*.

[14] Huang J., Chunlin L. (2006). Traditional cultivation of Coptis teeta and its values in biodiversity conservation. *Biodiversity Science*.

[15] Xu R., Chen J., Chen S. (2009). Medicinal plant resources achieve rational development and sustainable using. *China Pharmaceuticals*.

[16] Luqi H., Lanping G., Guanghong C., Peigen X., Yongyan W. (2005). Basic theory of traditional Chinese medicine resources. *Research & Information of Traditional Chinese Medicine*.

[17] Yang Y., Ma Y., Yang Z. (2012). Current status and development of cultivation of wild medicinal herbs. *World Science and Technology (Modernization of Traditional Chinese Medicine and Materia Medica)*.

[18] Wang H., Chen S. (2009). On the implementation of good manufacturing practices and pest control of herbal medicines. *Lishizhen Medicine and Materia Medica Research*.

[19] Gao W.-W., Zhao Y.-J., Wang Y.-P., Chen S.-L. (2006). A review of research on sustainable use of medicinal plants cropland in China. *China Journal of Chinese Materia Medica*.

[20] Yang Y.-H., Dai L.-J., He K.-H. (2012). Relation between soil nutrient of artificially cultivated area and rhizome quality of Paris polyphylla var. yunnanensis. *Journal of Chinese Medicinal Materials*.

[21] Han X.-L., Zhang X.-B., Guo L.-P. (2008). Statistical analysis of residues of heavy metals in Chinese crude drugs. *China Journal of Chinese Materia Medica*.

[22] Chen S., Jin S. (2002). A preliminary discussion on prevention and control of contamination of heavy metals and pesticides in Chinese medicinal plants. *World Science and Technology (Modernization of 
Traditional Chinese Medicine and Materia Medica)*.

[23] Wu F., Li M., Wang B. (2010). Experimental Study on the Resistance in *Fructus cannabis* Germplasm. *Journal of Anhui Agricultural Sciences*.

[24] Zhang T.-J. (2011). Realization and evaluation of Chinese materia medica quality. *Chinese Traditional and Herbal Drugs*.

[25] Chen S., Wei J., Sun C. (2006). Development of TCMGIS-I and its application in suitable producing area evaluation of Astragalus membranaceus. *World Science and Technology (Modernization of 
Traditional Chinese Medicine)*.

[26] Li X.-W., Chen S.-L. (2007). Conspectus of ecophysiological study on medicinal plant in wild nursery. *China Journal of Chinese Materia Medica*.

[27] Guodong H., Lanping G., Luqi H. (2008). Specialties and measures on variety breeding of chinese medicinal materials. *Resources Science*.

[28] Li X., Lin C., Li G. (2013). Influence of enclosure on *Glyeyrrhiza uralensis* community and distribution pattern in arid and semi-arid areas. *Acta Ecologica Sinica*.

[29] Shi Z.-Y., Bao Z., Jiang Y., Tu P.-F. (2007). Quantitative analysis of calycosin glycoside and formononetin in Radix Astragali from different sources. *China Journal of Chinese Materia Medica*.

[30] Li J., Li Y., Tai X. (2009). On the rural households livelihood in the western poor areas after the slopping land conversion program within the sustainable livelihood analysis framework from the rural households survey in the Zhouzhi County, Shanxi Province. *China Rural Survey*.

[31] Wang Z. (2006). Analysis of key issues which need to be resolved in the development of Chinese medicine industry. *China Journal of Chinese Materia Medica*.

[32] Peijun R. M. B., Yan M., Xiaohua W. (2010). Demonstration of standardized cultivation technology of Pinellia ternate. *Guizhou Agricultural Sciences*.

[33] Li M., Quan Y., Quan L. (2007). The progress and analysis of Panax ginseng cultivation in Yanbian State. *Journal of Agricultural Science Yanbian University*.

[34] Ministry of Finance of the People Public of China (2013). Developments of Chinese herbal undergrowth financial which support by Sichuan Province make sense. *Country Finance and Financial Fair*.

[35] Schippmann U. W. E., Leaman D., Cunningham A. B. A. (2006). Comparison of cultivation and wild collection of medicinal and aromatic plants under sustainability aspects. *Frontis*.

[36] Sun H., Jiang S.-Y., Feng C.-Q. (2012). Status of wild resource of medicine plant Lamiophlomis rotata and its problems in sustainable use. *China Journal of Chinese Materia Medica*.

[37] Rong W., Guo H., Yang H. (2006). Current research status in China on pesticide contamination of plant material used in making Chinese herbal medicines. *Agrochemicals*.

[38] Yi X., Lu Y. (2004). Study on residues of pesticides and heavy metals in ligusticum wallichii Franch and other seven kinds of traditional Chinese medicine. *Research and Practice of Chinese Medicines*.

[39] Wei S.-L., Wang W.-Q., Wang H. (2003). Study on licorice resources and their sustainable utilization in center and western area of China. *China Journal of Chinese Materia Medica*.

[40] Li X., Chen S. (2009). Study on conditions of worldwide natural protected areas and construction of medicinal function and ecological industry protected areas in China. *Journal of Natural Resources*.

[41] Liqun W., Ye X., Fen D. (2004). Quality assessment for Coptis chinensis planted with ecological techniques. *Acta Academiae Medicinae Sinicae*.

[42] Guifang C., Cheng R. (2003). A preliminary study on the effect of growing Coptis Chinensis to ecological environment. *Yunnan Geographic Environment Research*.

[43] Xiao P., Zhao R., Long X., Guo B. (2009). Macroscopic analysis on production and marketing of medicinal material resources for sustainable development. *China Journal of Chinese Materia Medica*.

[44] Wang J., Wang R., Fan S. (2012). Resources survey and analysis of wild *Forsythia suspensa* (Thunb.) Vahl. *Journal of Anhui Agricultural Sciences*.

[45] Ying X. (2006). The marketing analysis of Bupleurum chinense DC. *Modern Chinese Medicine*.

[46] Yang B., Li S., Wang X. (2008). On Cultivation and Rational Utilization of Paris polyphylla var. yunnanensis. *Chinese Wild Plant Resources*.

[47] Xiang D. (2010). The new investigation of Atractylodeschinensis (DC.) Koidz in Dong Bei. *Special Economic Animal and Plant*.

[48] Ding L. (2012). The producing and selling analysis of *Saposhnikovia divaricata* (Turcz.) Schischk. *Modern Chinese Medicine*.

[49] Yongwei Z., Fusheng J., Yin W., Zhishan D. (2012). Present status and sustainable development of Rhizoma Bletillae industry. *Chinese Archives of Traditional Chinese Medicine*.

[50] Chen S.-L., Jia M.-R., Wang Y., Xue G., Xiao P.-G. (2003). Study on the plant community of Fritillaria cirrhosa. *China Journal of Chinese Materia Medica*.

[51] Yang H. (2005). *Effect of the Grassland Enclosure on the Recovery of Gression Grassland and Field Liquorice Resource in Ningxia*.

[52] Hong H., Chen H., Xu F. (2011). Surveys on resources and varieties on Chinese markets of crude drug Mahuang. *China Journal of Chinese Materia Medica*.

[53] Zefeng L., Kun L., Yunliang H. (2011). The development status and tactics of Schisandra chinensis (Turcz.) Baill. industry in Liaoning Province. *Agricultural Economy*.

[54] Han W., Cao L., Hamid Y., Xu X. (2012). Adaptation of Apocynum Venetum to saline water irrigation. *Journal of Desert Research*.

[55] Cui L., Tong Q., Liang C., Wang C., Li Q. (2011). Research progress of environmental factors on growth and medicinal components of Acanthopanax. *Northern Horticulture*.

[56] Shengbo X., Jianju Q. (2013). Analyses on the types, distributions andcharacteristics of vegetation and soil along Qinghai-Tibet Railway. *Journal of Anhui Agricultural Sciences*.

[57] Chen S.-L., Wei J.-H., Huang L.-F., Guo B.-L., Xiao P.-G. (2004). Probing into the theory and practice of wild medicinal materials tending. *China Journal of Chinese Materia Medica*.

[58] Chunlian D., Xiaowu Z., Zhai L., Xiaopeng S. (2012). Hill County, Hebei Province, imitation of wild and cultivated Polyporus umbellatus (Pers.) Fries success in a sexual reproduction. *Edible and Medicinal Mushrooms*.

[59] Maoxiong R., Desheng W., Jianling Z. (2002). Wild resources of Epimedium brevicornum Maxim. with standardized planting and tending research. *The Chinese Academic Medical Magazine of Organisms*.

[60] Liqun Y., Wanlong D., Dianlong Z. (2008). Research advances on the wild medicinal materials tending and purposive cultivation of Trollius chinensis Bge. *Lishizhen Medicine and Materia Medica Research*.

[61] Liu Y., Liu Z., Xiao B. (2010). Wildlife tending of endangered herbal plant Dysosina versipellis. *Chinese Agricultural Science Bulletin*.

[62] Guoqing Y. (2009). The history of 8 Chinese medicine in Henan. *Lishizhen Medicine and Materia Medica Research*.

[63] Jihong H., Guoyan T. (2002). Advances in studies of Snow Lotuses (Saussurea). *Journal of Xinjiang Agricultural University*.

[64] Bahargul A.,  Xu Y., Guo Q., Gulibahaer, Rebiyaguli, Gulibositan (2013). Cistanche tubulosa resources, investigation and analysis of trade and cultivation. *Chinese Wild Plant Resources*.

[65] Hongmei Y., Qingqian C., Qianlie (2010). Set of Sarcandra glabra (Thunb.) Nakai cultivation techniques. *Hunan Forestry Science & Technology*.

